# 2-Nitro-*N*′-[1-(pyridin-2-yl)ethyl­idene]benzohydrazide

**DOI:** 10.1107/S1600536811049257

**Published:** 2011-11-19

**Authors:** Xiaofeng Li, Yan An, Yiqing Chen, Lishen Chen

**Affiliations:** a Institute of Marine Materials Science and Engineering, Shanghai Maritime University, Shanghai 201306, People’s Republic of China

## Abstract

In the title compound, C_14_H_12_N_4_O_3_, the dihedral angle between the benzene ring and the pyridine ring is 60.9 (2)°. The major twist in the mol­ecule occurs about the (NH)—(CO)—C_ar_—C_ar_ (ar = aromatic) bond, the relevant torsion angle being 63.97 (12)°. In the crystal, inversion dimers linked by pairs of N—H⋯O hydrogen bonds generate *R*_2_^2^(8) loops.

## Related literature

For related structures, see: Mangalam *et al.* (2009 ▶); Tang (2011 ▶).
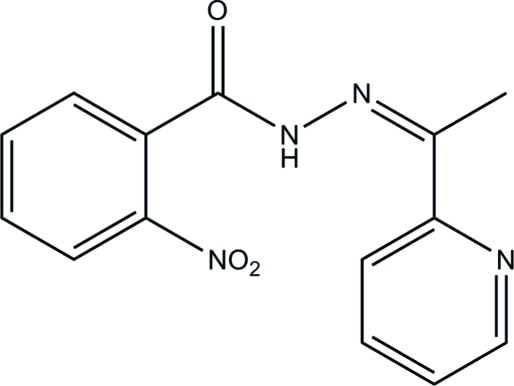


## Experimental

### 

#### Crystal data

C_14_H_12_N_4_O_3_
*M* *_r_* = 284.28
Monoclinic, 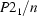

*a* = 10.8303 (8) Å
*b* = 8.9112 (7) Å
*c* = 14.9437 (11) Å
β = 101.483 (1)°
*V* = 1413.36 (18) Å^3^
*Z* = 4
Mo *K*α radiation
μ = 0.10 mm^−1^
*T* = 298 K
0.20 × 0.20 × 0.18 mm

#### Data collection

Bruker SMART 1K CCD diffractometer
Absorption correction: multi-scan (*SADABS*; Sheldrick, 1996 ▶) *T*_min_ = 0.981, *T*_max_ = 0.983
7984 measured reflections
3048 independent reflections
2358 reflections with *I* > 2σ(*I*)
*R*_int_ = 0.015

#### Refinement

*R*[*F* ^2^ > 2σ(*F* ^2^)] = 0.043
*wR*(*F* ^2^) = 0.126
*S* = 1.05
3048 reflections
194 parameters
1 restraint
H atoms treated by a mixture of independent and constrained refinement
Δρ_max_ = 0.23 e Å^−3^
Δρ_min_ = −0.19 e Å^−3^

### 

Data collection: *SMART* (Bruker, 2007 ▶); cell refinement: *SAINT* (Bruker, 2007 ▶); data reduction: *SAINT*; program(s) used to solve structure: *SHELXS97* (Sheldrick, 2008 ▶); program(s) used to refine structure: *SHELXL97* (Sheldrick, 2008 ▶); molecular graphics: *SHELXTL* (Sheldrick, 2008 ▶); software used to prepare material for publication: *SHELXTL*.

## Supplementary Material

Crystal structure: contains datablock(s) I, global. DOI: 10.1107/S1600536811049257/hb6488sup1.cif

Structure factors: contains datablock(s) I. DOI: 10.1107/S1600536811049257/hb6488Isup2.hkl

Supplementary material file. DOI: 10.1107/S1600536811049257/hb6488Isup3.cml

Additional supplementary materials:  crystallographic information; 3D view; checkCIF report

## Figures and Tables

**Table 1:** Hydrogen-bond geometry (Å, °)

*D*—H⋯*A*	*D*—H	H⋯*A*	*D*⋯*A*	*D*—H⋯*A*
N3—H3⋯O1^i^	0.90 (1)	2.13 (1)	3.0290 (15)	173 (2)

Symmetry code: (i) 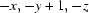.

**Fig. 1.**
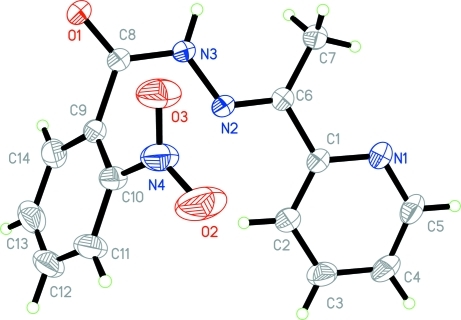
The molecular structure of the title compound, with the displacement ellipsoids drawn at the 30% probability level.

**Fig. 2.**
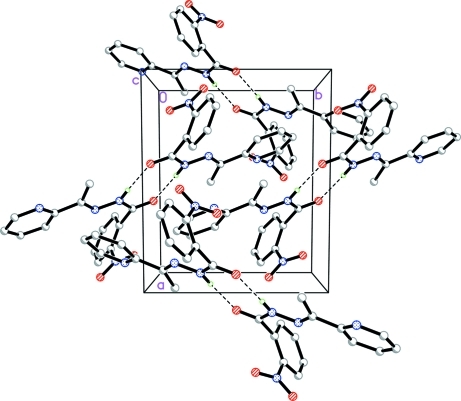
The molecular packing of the title compound, viewed along the c axis. Intermolecular N—H···O hydrogen-bonds are shown as dashed lines. H-atoms not involved in the hydrogen bonding have been omitted.

## supplementary crystallographic information

### Comment

As a continuation of the work on the structures of hydrazone compounds the title compound, (I), is now described.

The molecular structure of the title compound is shown as Fig. 1. The dihedral angle between the benzene ring and the pyridine ring is 60.9 (2)°, indicating the molecule of the compound is much distorted. The bond distances comparable to the values observed in similar compounds (Tang, 2011; Mangalam *et al.*, 2009).

In the crystal structure of the compound, adjacent two molecules are linked through two intermolecular N—H···O hydrogen bonds (Table 1) to form a dimer (Fig. 2).

### Experimental

Equimolar quantities (0.5 mmol each) of 2-acetylpyridine and 2-nitrobenzohydrazide were mixed in 30 ml me thanol. The mixture was stirred at reflux for 30 min and cooled to room temperature. Yellow block-shaped single crytals were formed by slow evaporation of the solvent in air.

### Refinement

H3 atom was located in a difference Fourier map and was refined with distance restraint, N—H = 0.90 (1) Å. The remaining H atoms were positioned geometrically and refined using a riding model, with C—H = 0.93–0.96 Å, and with *U*_iso_(H) = 1.2*U*_eq_(C) and 1.5*U*_eq_(C7).

### Crystal data

**Table:**

C_14_H_12_N_4_O_3_	*F*(000) = 592
*M**_r_* = 284.28	*D*_x_ = 1.336 Mg m^−^^3^
Monoclinic, *P*2_1_/*n*	Mo *K*α radiation, λ = 0.71073 Å
Hall symbol: -P 2yn	Cell parameters from 3300 reflections
*a* = 10.8303 (8) Å	θ = 2.6–28.3°
*b* = 8.9112 (7) Å	µ = 0.10 mm^−^^1^
*c* = 14.9437 (11) Å	*T* = 298 K
β = 101.483 (1)°	Block, yellow
*V* = 1413.36 (18) Å^3^	0.20 × 0.20 × 0.18 mm
*Z* = 4	

### Data collection

**Table:**

Bruker SMART 1K CCD diffractometer	3048 independent reflections
Radiation source: fine-focus sealed tube	2358 reflections with *I* > 2σ(*I*)
graphite	*R*_int_ = 0.015
ω scan	θ_max_ = 27.0°, θ_min_ = 2.1°
Absorption correction: multi-scan (*SADABS*; Sheldrick, 1996)	*h* = −13→9
*T*_min_ = 0.981, *T*_max_ = 0.983	*k* = −11→11
7984 measured reflections	*l* = −18→19

### Refinement

**Table:**

Refinement on *F*^2^	Primary atom site location: structure-invariant direct methods
Least-squares matrix: full	Secondary atom site location: difference Fourier map
*R*[*F*^2^ > 2σ(*F*^2^)] = 0.043	Hydrogen site location: inferred from neighbouring sites
*wR*(*F*^2^) = 0.126	H atoms treated by a mixture of independent and constrained refinement
*S* = 1.05	*w* = 1/[σ^2^(*F*_o_^2^) + (0.0611*P*)^2^ + 0.2704*P*] where *P* = (*F*_o_^2^ + 2*F*_c_^2^)/3
3048 reflections	(Δ/σ)_max_ < 0.001
194 parameters	Δρ_max_ = 0.23 e Å^−^^3^
1 restraint	Δρ_min_ = −0.19 e Å^−^^3^

### Special details

**Table:**

Geometry. All e.s.d.'s (except the e.s.d. in the dihedral angle between two l.s. planes) are estimated using the full covariance matrix. The cell e.s.d.'s are taken into account individually in the estimation of e.s.d.'s in distances, angles and torsion angles; correlations between e.s.d.'s in cell parameters are only used when they are defined by crystal symmetry. An approximate (isotropic) treatment of cell e.s.d.'s is used for estimating e.s.d.'s involving l.s. planes.
Refinement. Refinement of *F*^2^ against ALL reflections. The weighted *R*-factor *wR* and goodness of fit *S* are based on *F*^2^, conventional *R*-factors *R* are based on *F*, with *F* set to zero for negative *F*^2^. The threshold expression of *F*^2^ > σ(*F*^2^) is used only for calculating *R*-factors(gt) *etc*. and is not relevant to the choice of reflections for refinement. *R*-factors based on *F*^2^ are statistically about twice as large as those based on *F*, and *R*- factors based on ALL data will be even larger.

### Fractional atomic coordinates and isotropic or equivalent isotropic displacement parameters (Å^2^)

**Table:**

	*x*	*y*	*z*	*U*_iso_*/*U*_eq_	
N1	0.12218 (13)	1.12660 (15)	0.19162 (9)	0.0549 (3)	
N2	0.13344 (11)	0.83590 (12)	0.03440 (8)	0.0444 (3)	
N3	0.09007 (12)	0.69373 (13)	0.00993 (8)	0.0476 (3)	
N4	0.39424 (14)	0.72128 (18)	0.02669 (11)	0.0702 (4)	
O1	0.09268 (11)	0.50167 (12)	−0.08683 (8)	0.0591 (3)	
O2	0.47052 (19)	0.8063 (2)	0.07159 (12)	0.1265 (8)	
O3	0.36144 (14)	0.60537 (16)	0.05665 (10)	0.0845 (4)	
C1	0.14756 (12)	1.05765 (15)	0.11797 (9)	0.0416 (3)	
C2	0.21742 (15)	1.12613 (17)	0.06083 (11)	0.0535 (4)	
H2	0.2356	1.0747	0.0109	0.064*	
C3	0.25946 (17)	1.27091 (18)	0.07897 (13)	0.0629 (4)	
H3A	0.3046	1.3194	0.0407	0.075*	
C4	0.23368 (17)	1.34274 (18)	0.15460 (12)	0.0631 (5)	
H4	0.2610	1.4404	0.1686	0.076*	
C5	0.16671 (18)	1.26654 (19)	0.20863 (12)	0.0641 (5)	
H5	0.1510	1.3147	0.2604	0.077*	
C6	0.09516 (13)	0.90373 (15)	0.09935 (9)	0.0416 (3)	
C7	0.00521 (17)	0.8435 (2)	0.15426 (12)	0.0616 (4)	
H7A	−0.0685	0.8058	0.1140	0.092*	
H7B	−0.0185	0.9222	0.1914	0.092*	
H7C	0.0448	0.7637	0.1928	0.092*	
C8	0.13068 (14)	0.62602 (15)	−0.05927 (10)	0.0452 (3)	
C9	0.21832 (14)	0.71408 (15)	−0.10665 (10)	0.0460 (3)	
C10	0.33825 (15)	0.76293 (16)	−0.06705 (11)	0.0519 (4)	
C11	0.41041 (18)	0.8496 (2)	−0.11371 (14)	0.0666 (5)	
H11	0.4896	0.8832	−0.0848	0.080*	
C12	0.3637 (2)	0.8853 (2)	−0.20298 (15)	0.0751 (6)	
H12	0.4120	0.9420	−0.2354	0.090*	
C13	0.2465 (2)	0.8381 (2)	−0.24480 (14)	0.0759 (6)	
H13	0.2152	0.8628	−0.3055	0.091*	
C14	0.17386 (18)	0.7532 (2)	−0.19668 (11)	0.0611 (4)	
H14	0.0939	0.7221	−0.2257	0.073*	
H3	0.0368 (15)	0.640 (2)	0.0371 (13)	0.080*	

### Atomic displacement parameters (Å^2^)

**Table:**

	*U*^11^	*U*^22^	*U*^33^	*U*^12^	*U*^13^	*U*^23^
N1	0.0659 (8)	0.0473 (7)	0.0524 (7)	−0.0064 (6)	0.0134 (6)	−0.0128 (6)
N2	0.0521 (7)	0.0348 (6)	0.0491 (7)	−0.0093 (5)	0.0172 (5)	−0.0071 (5)
N3	0.0570 (7)	0.0385 (6)	0.0534 (7)	−0.0140 (5)	0.0257 (6)	−0.0098 (5)
N4	0.0666 (9)	0.0687 (10)	0.0739 (10)	−0.0229 (8)	0.0109 (8)	0.0082 (8)
O1	0.0779 (8)	0.0422 (6)	0.0646 (7)	−0.0196 (5)	0.0320 (6)	−0.0173 (5)
O2	0.1304 (14)	0.1325 (16)	0.0997 (12)	−0.0800 (12)	−0.0180 (11)	0.0141 (11)
O3	0.0905 (10)	0.0725 (9)	0.0852 (9)	−0.0211 (7)	0.0049 (7)	0.0280 (7)
C1	0.0429 (7)	0.0365 (7)	0.0433 (7)	−0.0008 (5)	0.0038 (5)	−0.0034 (5)
C2	0.0646 (9)	0.0412 (8)	0.0570 (9)	−0.0072 (7)	0.0176 (7)	−0.0042 (7)
C3	0.0719 (11)	0.0439 (9)	0.0733 (11)	−0.0115 (7)	0.0154 (9)	0.0046 (8)
C4	0.0726 (11)	0.0378 (8)	0.0733 (11)	−0.0093 (7)	0.0007 (9)	−0.0071 (8)
C5	0.0777 (11)	0.0503 (9)	0.0618 (10)	−0.0057 (8)	0.0077 (8)	−0.0203 (8)
C6	0.0445 (7)	0.0398 (7)	0.0410 (7)	−0.0042 (5)	0.0094 (5)	−0.0038 (5)
C7	0.0698 (10)	0.0616 (10)	0.0604 (10)	−0.0224 (8)	0.0298 (8)	−0.0164 (8)
C8	0.0539 (8)	0.0367 (7)	0.0481 (8)	−0.0081 (6)	0.0179 (6)	−0.0062 (6)
C9	0.0601 (8)	0.0325 (7)	0.0516 (8)	−0.0031 (6)	0.0258 (7)	−0.0049 (6)
C10	0.0611 (9)	0.0394 (8)	0.0607 (9)	−0.0077 (6)	0.0255 (7)	0.0005 (7)
C11	0.0694 (11)	0.0533 (10)	0.0869 (13)	−0.0105 (8)	0.0394 (10)	0.0058 (9)
C12	0.0996 (15)	0.0567 (11)	0.0852 (13)	−0.0009 (10)	0.0576 (12)	0.0125 (9)
C13	0.1131 (17)	0.0669 (12)	0.0576 (10)	0.0116 (11)	0.0404 (11)	0.0133 (9)
C14	0.0751 (11)	0.0581 (10)	0.0540 (10)	0.0025 (8)	0.0225 (8)	−0.0023 (7)

### Geometric parameters (Å, °)

**Table:**

N1—C1	1.3360 (18)	C4—H4	0.9300
N1—C5	1.343 (2)	C5—H5	0.9300
N2—C6	1.2805 (17)	C6—C7	1.493 (2)
N2—N3	1.3752 (15)	C7—H7A	0.9600
N3—C8	1.3446 (18)	C7—H7B	0.9600
N3—H3	0.903 (9)	C7—H7C	0.9600
N4—O3	1.2071 (19)	C8—C9	1.5118 (19)
N4—O2	1.219 (2)	C9—C14	1.380 (2)
N4—C10	1.459 (2)	C9—C10	1.386 (2)
O1—C8	1.2245 (16)	C10—C11	1.382 (2)
C1—C2	1.390 (2)	C11—C12	1.367 (3)
C1—C6	1.4892 (19)	C11—H11	0.9300
C2—C3	1.377 (2)	C12—C13	1.365 (3)
C2—H2	0.9300	C12—H12	0.9300
C3—C4	1.375 (3)	C13—C14	1.391 (3)
C3—H3A	0.9300	C13—H13	0.9300
C4—C5	1.368 (3)	C14—H14	0.9300
			
C1—N1—C5	117.26 (14)	C6—C7—H7B	109.5
C6—N2—N3	119.44 (11)	H7A—C7—H7B	109.5
C8—N3—N2	118.11 (11)	C6—C7—H7C	109.5
C8—N3—H3	116.5 (13)	H7A—C7—H7C	109.5
N2—N3—H3	125.4 (13)	H7B—C7—H7C	109.5
O3—N4—O2	123.02 (17)	O1—C8—N3	121.68 (13)
O3—N4—C10	118.49 (14)	O1—C8—C9	120.83 (12)
O2—N4—C10	118.48 (15)	N3—C8—C9	117.34 (11)
N1—C1—C2	122.05 (13)	C14—C9—C10	116.89 (14)
N1—C1—C6	116.38 (12)	C14—C9—C8	117.24 (14)
C2—C1—C6	121.56 (12)	C10—C9—C8	125.84 (14)
C3—C2—C1	119.31 (15)	C11—C10—C9	122.42 (16)
C3—C2—H2	120.3	C11—C10—N4	117.24 (15)
C1—C2—H2	120.3	C9—C10—N4	120.33 (13)
C4—C3—C2	119.00 (16)	C12—C11—C10	119.04 (18)
C4—C3—H3A	120.5	C12—C11—H11	120.5
C2—C3—H3A	120.5	C10—C11—H11	120.5
C5—C4—C3	118.18 (15)	C13—C12—C11	120.35 (17)
C5—C4—H4	120.9	C13—C12—H12	119.8
C3—C4—H4	120.9	C11—C12—H12	119.8
N1—C5—C4	124.17 (16)	C12—C13—C14	120.08 (18)
N1—C5—H5	117.9	C12—C13—H13	120.0
C4—C5—H5	117.9	C14—C13—H13	120.0
N2—C6—C1	114.05 (12)	C9—C14—C13	121.19 (18)
N2—C6—C7	126.30 (12)	C9—C14—H14	119.4
C1—C6—C7	119.65 (12)	C13—C14—H14	119.4
C6—C7—H7A	109.5		

### Hydrogen-bond geometry (Å, °)

**Table:**

*D*—H···*A*	*D*—H	H···*A*	*D*···*A*	*D*—H···*A*
N3—H3···O1^i^	0.90 (1)	2.13 (1)	3.0290 (15)	173.(2)

Symmetry codes: (i) −*x*, −*y*+1, −*z*.
